# Evolving partnerships: engagement methods in an established health services research team

**DOI:** 10.1186/s40900-021-00314-w

**Published:** 2021-10-09

**Authors:** Stephanie A. Chamberlain, Andrea Gruneir, Janice M. Keefe, Charlotte Berendonk, Kyle Corbett, Roberta Bishop, Graham Bond, Faye Forbes, Barbara Kieloch, Jim Mann, Christine Thelker, Carole A. Estabrooks

**Affiliations:** 1 Department of Family Medicine, Faculty of Medicine and Dentistry, University of Alberta, Edmonton, Alberta Canada; 2 Department of Family Studies and Gerontology, Mount Saint Vincent University, Halifax, Nova Scotia Canada; 3 Faculty of Nursing, University of Alberta, Edmonton, Alberta Canada; 4 Department of Medicine, Faculty of Medicine and Dentistry, University of Alberta, Edmonton, Alberta Canada; 5 Voices Of Individuals, family and friend Caregivers Educating uS (VOICES), University of Alberta, Edmonton, Alberta Canada

**Keywords:** Citizen engagement, Health services research, Engagement science, Integrated knowledge translation

## Abstract

**Background:**

The Translating Research in Elder Care (TREC) program is a partnered health services research team that aims to improve the quality of care and quality of life for residents and quality of worklife for staff in nursing homes. This team includes academic researchers, trainees, research staff, citizens (persons living with dementia and family/friend caregivers of persons living in nursing homes), and decision-makers (ministries of health, health authorities, operators of nursing homes). The TREC team has experience working with health system partners but wanted to undertake activities to enhance the collaboration between the academic researchers and citizen members. The aim of this paper is to describe the TREC team members’ experiences and perceptions of citizen engagement and identify necessary supports to promote meaningful engagement in health research teams.

**Methods:**

We administered two online surveys (May 2018, July 2019) to all TREC team members (researchers, trainees, staff, decision-makers, citizens). The surveys included closed and open-ended questions guided by regional and international measures of engagement and related to respondents’ experience with citizen engagement, their perceptions of the benefits and challenges of citizen engagement, and their needs for training and other tools to support engagement. We analyzed the closed-ended responses using descriptive statistics.

**Results:**

We had a 78% response rate (68/87) to the baseline survey, and 27% response rate (21/77) to the follow-up survey. At baseline, 30 (44%) of respondents reported they were currently engaged in a research project with citizen partners compared to 11(52%) in the follow-up survey. Nearly half (10(48%)) of the respondents in the follow-up reported an increase in citizen engagement over the previous year. Respondents identified many benefits to citizen engagement (unique perspectives, assistance with dissemination) and challenges (the need for specific communication skills, meeting organizing and facilitation, and financial/budget support), with little change between the two time points. Respondents reported that the amount of citizen engagement in their research (or related projects) had increased or stayed the same.

**Conclusions:**

Despite increasing recognition of the benefits of including persons with lived experience and large-scale promotion efforts, the research team still lack sufficient training and resources to engage non-academic partners. Our research identified specific areas that could be addressed to improve the engagement of citizens in health research.

**Supplementary Information:**

The online version contains supplementary material available at 10.1186/s40900-021-00314-w.

## Plain English Summary

Engaging non-academic research partners is becoming an expectation for Canadian health research teams. Research specifically on citizen (patient) engagement has identified benefits and challenges of partnered research; however, most research has only examined one time point. Less is known about how the perception of engagement may change with exposure to engagement in research activities. Translating Research in Elder Care (TREC) is a research program that aims to improve the quality of care and quality of life for residents, and quality of work life of staff in nursing homes. The team includes academic researchers, citizens (the term preferred by our members to reflect persons with dementia, family/friend caregivers of individuals living in nursing homes), decision-maker stakeholders (e.g., ministries of health, operators of nursing homes), trainees (Masters, PhD, postdoctoral fellows), and staff. The TREC team initiated several activities to deepen the partnerships with our citizen members. This paper describes the teams’ perceptions of engagement and the benefits and challenges of citizen engagement in an established health research team. We invited the TREC team to complete an online survey (May 2018) before undertaking engagement activities (e.g., training, priority setting) and after 15 months of activities (July 2019). We asked respondents questions about their experience with citizen engagement, their perceptions of the potential research activities citizens could be engaged in, and the benefits and challenges of citizen engagement. Research team respondents reported an increase in citizen engagement in their research but that needs for support persisted. We identify specific areas where research teams need more training and support to ensure that engaged research is possible and sustainable.

**Supplementary Information:**

The online version contains supplementary material available at 10.1186/s40900-021-00314-w.

## Introduction

Engagement science encompasses the methods, frameworks, and resources necessary to support and advance research conducted in partnership with non-academic stakeholders [1, 2]. Its emergence as a distinct scientific discipline illustrates how the various forms of partnered research, including action research, patient-oriented research, and community-engaged research, have been formalized in the larger health research ecosystem [3]. While the engagement of non-academics, such as clinicians or policy makers, is not new, the past decade has seen a substantial emphasis on the purposeful engagement of individuals with lived experience, be it patients, clients, caregivers, or others [4–6]. Major international initiatives including the Patient Centered Outcomes Research Institute (PCORI) in the United States, INVOLVE in the United Kingdom, and the Strategy for Patient-Oriented Research (SPOR) in Canada, have served as the foundation for this movement and have promoted engagement from a unique selling point to an expectation of health research [1, 7–9].

Engagement has benefits and challenges [10–12]. Engagement is thought to lead to better, more relevant research questions, increased recruitment, broader and more direct dissemination, and better alignment of research and end-user needs [2, 11, 12]. Health researchers, in particular those in clinical and applied fields, report generally positive perceptions of engagement despite concerns about their own understanding of the methods of engagement and its implementation [13]. Others have raised concerns about engagement being a trend, identified challenges in recruitment, cited the limited evidence on benefits of engagement to the research process, and highlighted the lack of research on the influence of engagement on research outcomes [2, 14]. Much of this research on the attitudes and perceptions of engagement in research has been cross-sectional. While these reports have helped to characterize the perceived benefits and concerns held by researchers and other research partners, none have yet described how these perceptions may change with greater exposure to engagement in their research activities.

The Translating Research in Elder Care (TREC) program, established in 2007, is a multi-disciplinary team of researchers and non-academic stakeholders working to improve the care of older adults living in nursing homes [15–17]. In 2016, TREC established a citizen (preferred by members to the word “patient” that is more closely associated with acute care/hospital settings) advisory committee called VOICES, comprised of anywhere between 10 and 12 persons living with dementia and/or family/friend caregivers to someone living in a nursing home [16]. The TREC research team (researchers, trainees, staff) were accustomed to working with health system decision makers (e.g., provincial policy makers, owner-operators of nursing homes), but the inclusion of citizens—and the different knowledge that they brought to the team—was new. Over the course of 15-months (May 2018–July 2019), TREC senior leadership, based on input from VOICES members, decided to undertake several activities intended to deepen team engagement and collaboration. Activities were decided in collaboration between VOICES and TREC leadership, some of which were directly funded through an external grant [16]. Activities aimed to provide basic training on engagement, integration of VOICES at meetings and on other committees, and identify their priorities for analysis. Specific activities we undertook included (1) a full team (including researchers, trainees, staff, decision-makers, citizens) day-long training workshop on citizen engagement, (2) the introduction of internal TREC policies to include VOICES members on individual research projects, (3) increasing the presence of VOICES members at all TREC researcher meetings and purposeful inclusion on these meeting agendas, and (4) an internal priority setting project to guide the secondary analysis of TREC’s data resources [16]. The full team day-long training was facilitated by the Alberta SPOR Support Unit Patient Engagement Platform [8]. The training provided an overview of citizen-oriented research, the research cycle and related research activities, and a number of small and large group activities and discussions about engaging citizens in the team. We sought to understand TREC team members’ experiences with and perceptions of engagement both before and after these activities that were intended to promote more meaningful engagement.

## Methods

We administered two online surveys using a secure Canadian survey provider (https://simplesurvey.com/) to all members of the TREC team. The TREC team includes researchers, trainees (Masters and PhD students, postdoctoral fellows), staff (e.g., project managers, data analysts), collectively identified as the “research team”, and non-academic partners which includes decision makers (e.g., regional health authority, provincial ministry, nursing home operator) and citizens (VOICES). The surveys were distributed at two time points. The first survey was sent prior to the team engagement training (May 2018). The second survey was sent after the conclusion of the various engagement activities (July 2019). Each survey was followed by two reminder emails. Due to fluctuation in the team composition over the year (e.g., trainees graduating, decision-makers/staff leaving their positions), the total number of team members changed slightly between timepoints (see Table 1).

**Table 1:** Respondent roles

Role	Baseline, n (%)		Follow-up, n (%)	
	Sent to n = 87	Responded n = 68	Sent to n = 77	Responded n = 21
Researchers	34	17 (25)	31	8 (38)
Trainees	8	6 (9)	7	3 (14)
Staff	18	14 (21)	16	2 (10)
Decision-makers	16	7 (10)	14	3 (14)
VOICES	11	7 (10)	9	5 (24)
Missing role	–	17 (25)	–	0

The online surveys included closed and open-ended questions. The questions were informed by existing measures of engagement (e.g., IAP2), SPOR training related to the research process, and team interest in general benefits and challenges [8, 18]. The specific questions related to respondents’ experiences with citizen engagement, perceptions about the potential for citizen engagement at each stage of the research process (identifying the research question, designing the study, collecting the data, analyzing the data, disseminating the findings), perceptions of the benefits and challenges of citizen engagement, and need for training and tools to support engagement (see Supplementary File 1 for copies of each survey). We asked respondents about the depth of their experience with citizen engagement, asking them to identify current and previous level of engagement using the International Association of Public Participation (IAP2) spectrum of best practices in public engagement. The IAP2 spectrum ranges from the lowest level of engagement “inform” (which includes activities like letting patients/public know about the research findings) to “involve” (where patients/public work with researchers as partners) and “empower” (the highest level of engagement where patients/public actively control and direct the research process) [18]. Survey topics were decided upon by the team (including VOICES) but the final survey design and implementation was led by 3 members of the research team (AG, SC, CB), who did not respond to the surveys. Surveys were also piloted by a number of research team members (investigators, staff, trainees) to ensure usability and comprehension.

### Analysis

We used descriptive summary statistics to analyze the quantitative responses. Given the small sample size, we could not conduct more robust quantitative analyses of change over time. Where possible, we present results that are disaggregated by respondent type (i.e., research team, decision-maker, VOICES). Questions about the benefits, challenges, and need for support to conduct research with citizens had particular relevance to the research team and therefore only responses from this group are presented. Open-ended survey responses are presented to illustrate the quantitative findings. The quantitative analysis of the survey results was completed by the academic research team.

## Results

See Table 1 for the number of respondents to each survey. A total of 68 individuals responded to the baseline survey in May 2018 for a response rate of 78% (68/87). Of these 17 (25%) identified as researchers, 6 (9%) as trainees, 14 (21%) as TREC staff, 7 (10%) as decision makers, and 7 (10%) as VOICES members; 17 (25%) did not report their role on the team. We did not include those that did not report their role in subsequent analyses. For the follow-up survey, there were a total of 21 respondents, for a response rate of 27% (21/77), of whom 8 (38%) were researchers, 3 (14%) were trainees, 5 (24%) were VOICES members and the remainder were decision makers and TREC staff. For the purposes of our analysis, we combined the responses from researchers, staff, and trainees and described them as the “research team” because their primary role is the management and conduct of research activities. We present open-text survey responses from the research team to illustrate the survey findings. We did not have open-text responses from our citizen members because either none were provided, or the responses were single words.

### Experience with engagement

**Fig. 1:**
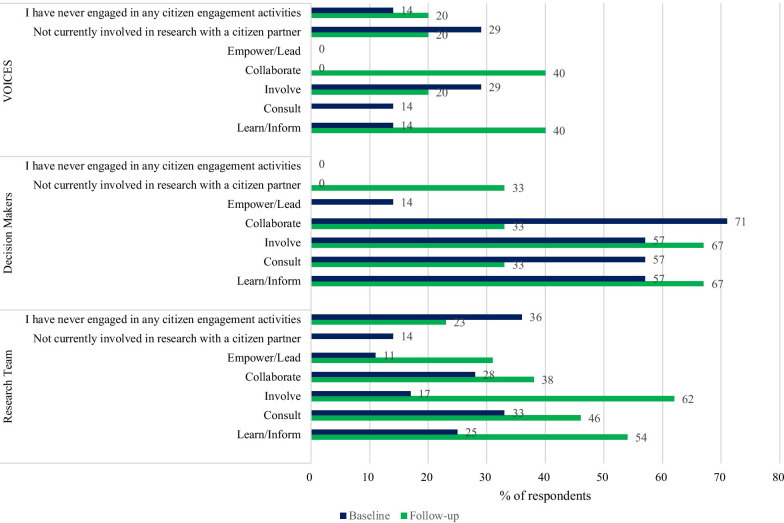
Engagement methods in current research projects by respondent type

Figure 1 illustrates how the respondent types (research team, decision-makers, VOICES members) reported their activity on the IAP2 spectrum of engagement [18]. Many respondents reported their that the activity was on the lower end of the spectrum with a greater proportion engaging in learn/inform/consult compared to collaborate/empower/lead (Fig. 1). At baseline, 44% (30/68) of all respondents reported that they were currently engaged in a research project with citizen partners.

When asked about the potential for citizens to play a role in each stage of the research process, Table 2 demonstrates that of the three respondent groups, the research team (investigators, staff, trainees) primarily saw a role for citizen partners in identifying the research questions and objectives (baseline: 30(83%), follow-up: 12(92%)) and dissemination (baseline: 28(78%), follow-up: 11(85%)) (Table 2). Respondents in the open-text described the importance of engaging citizen partners and soliciting their expertise before a grant is proposed to ensure that their interests shape the research questions. Decision-makers described how citizens are often well-connected advocates in the community and that their roles outside the research program could help to spread findings to non-academic audiences. There was greater uncertainty around citizen roles in areas such as study design, data collection or data analysis. The research team indicated concerns with incorporating citizens into roles that would require training or specific skills, such as data collection or analysis. Decision-maker respondents and VOICES also reported a role for citizens in identifying research questions and dissemination although there were few respondents who answered at follow-up.

**Table 2:** Roles for citizens in different stages of research

	Research Team		Decision Makers		VOICES Members	
	Baseline (n = 36) n, %	Follow-up (n = 13) n, %	Baseline (n = 7) n, %	Follow-up (n = 3) n, %	Baseline (n = 7) n, %	Follow-up (n = 5) n, %
*Identifying the research question(s) and objective(s)*						
Yes	30 (83)	12 (92)	6 (86)	3 (100)	6 (86)	5 (100)
No/don’t know	3 (8)	0	0	0	1 (14)	0
Missing	4 (11)	1 (8)	1 (14)	0	0	0
*Designing the study*						
Yes	25 (69)	9 (69)	5 (71)	2 (67)	2 (29)	1 (20)
No/don’t know	8 (22)	0	1 (14)	0	4 (57)	0
Missing	3 (8)	4 (31)	1 (14)	1 (33)	1 (14)	4 (80)
*Collecting the data*						
Yes	22 (61)	7 (54)	4 (57)	2 (67)	1 (14)	2 (40)
No/don’t know	10 (28)	0	2 (29)	0	5 (71)	0
Missing	4 (11)	6 (46)	1 (14)	1 (33)	1 (14)	3 (60)
*Analyzing the data*						
Yes	13 (36)	6 (46)	5 (71)	3 (100)	2 (29)	2 (40)
No/don’t know	18 (50)	0	1 (14)	0	4 (57)	0
Missing	5 (14)	7 (54)	1 (14)	0	1 (14)	3 (60)
*Disseminating the findings*						
Yes	28 (78)	11 (85)	5 (71)	3 (100)	4 (57)	4 (80)
No/don’t know	5 (14)	0	1 (14)	0	2 (29)	0
Missing	3 (8)	2 (15)	1 (14)	0	1 (14)	1 (20)

### Benefits and challenges

At both time points (baseline and follow-up), the research team reported benefits for citizen engagement in their research by highly rating the importance of citizens’ “unique perspective” (81% and 100%, respectively) and ability to help the team “generate new ideas” (86% and 100%, respectively). A single respondent reported no benefit to citizen partnered research at follow-up. At follow-up, the research team respondents reported a higher rating in each of the categories of benefits of engagement. When asked about other benefits of citizen engagement, respondents’ comments focused on improving research relevance and usability and adding meaning to the work, exemplified by statements such as:

“They remind researchers of the reasons why we do the work we do.” (Researcher, baseline)

“They make research more meaningful” (Researcher, baseline)

We asked the research team to identify the benefits, challenges, support needs, and skills to conduct citizen oriented research (Table 3). The challenges reported by the research team of citizen engagement in research related to practical concerns, namely: identifying citizen partners (64% baseline and 77% follow-up), time (58% and 77%, respectively), and cost (50% and 54%, respectively) (Table 3). Approximately one-quarter (22% and 23%, respectively) of the research team indicated they were unclear what activities citizen partners could be engaged in. Other concerns, such as loss of control or impact on scientific rigour were infrequently described at either time point. Other challenges noted by respondents included the skills and readiness for researchers to take on meaningful engagement:

“Current attitude/aptitude of many researchers is a major barrier” (Staff, baseline)

**Table 3:** Research team perception on the benefits, challenges, support needs, and skills to conduct citizen engaged research

	Baseline (n = 36)	Follow-up (n = 13)
*Benefits of citizen engaged research*		
Satisfy requirements for funding agencies (e.g., Canadian Institutes of Health Research)	23 (64)	12 (92)
Unique perspectives	29 (81)	13 (100)
Support for knowledge translation	26 (72)	13 (100)
Generate new ideas	31 (86)	13 (100)
Provide connections to other relevant groups (other citizen groups, decision-makers, front line staff)	30 (83)	12 (92)
Access/support for data collection (e.g., connection to new sites, assist in recruiting participants)	25 (69)	8 (62)
I do not see a benefit to partnered research	0	1 (8)
Other	4 (11)	2 (15)
*Challenges of citizen engaged research*		
Time	21 (58)	10 (77)
Cost	18 (50)	7 (54)
Identifying citizen partners	23 (64)	10 (77)
Loss of control	5 (14)	1 (8)
Impact on scientific rigor	4 (11)	1 (8)
Unclear what activities partner could be engaged in	8 (22)	3 (23)
I do not see any barriers or challenges to partnered research	4 (11)	1 (8)
Other	7 (19)	2 (15)
*Support needed to conduct citizen engaged research*		
Communication with non-academic audiences (written materials)	17 (47)	4 (31)
Communication with non-academic audiences (verbal/presentation skills)	16 (44)	4 (31)
Meeting facilitation	14 (39)	5 (38)
Budget supports (how to budget for citizen partners)	18 (50)	9 (69)
Staff support	15 (42)	6 (46)
Other	7 (19)	0
*Skills to conduct citizen engaged research*		
Yes	14 (39)	10 (77)
No	3 (8)	0
Unsure	13 (36)	2 (15)
Missing	6 (16)	1 (8)

As well, the research team raised concerns about the potential overuse/overexposure of citizen members who are participating in multiple projects and programs of research:

“Concern when a small group of citizen partners are key consultants on multiple projects, both in terms of fatigue and rigor” (Researcher, baseline)

After a little over a year of engagement activities, respondent comments about the challenges of citizen engagement evolved to include a focus on better integrating partners and expectation management, exemplified by the following statement in the open-ended section.

“Managing expectations, avoiding tokenism, adequately identifying and accommodating citizens’ needs” (Researcher, follow-up)

### Need for support

When asked about support needed to conduct citizen engaged research, respondents to both the baseline and follow-up survey identified the need for communication skills (written and verbal), meeting facilitation, and staff support. Budget support was frequently cited in both surveys and included guidance on how to budget appropriately in grant proposals (e.g., remuneration standards, additional costs for non-academic partners).

When asked for other support needs, open-text responses focused on obtaining training (“could always use more training”) with multiple comments specifically coming back to the need for development in communication skills (“better communication skills and maintaining connections”) and ways to identify suitable citizen partners (“awareness of citizen groups and recruitment strategies”). In the follow-up survey, comments included the need for better training for researchers in study design (“how to involve citizen partners from the design/idea generation stage of a project”) as well as comments reflecting concerns about expectation management and the differing needs of partners (“colliding academic and non-academic worlds.”). One researcher noted needing specific strategies for communicating with citizens about the research process:

“[Need skills in the] best ways to keep them informed when the pace of research is slow (e.g., delays in getting funding, parts of the project where not all team members need to be active in the process” (Researcher, follow-up)

At follow-up, a higher proportion of research team members felt they had the skills to conduct citizen engaged research, compared to baseline (39% baseline, 77% follow-up). After a year of activities, two thirds (62%, n = 8) of the research team (researchers, trainees, staff) felt that their engagement with citizens had stayed the same and 38% (n = 5) said it increased. None of the respondents (n = 21) (research team, decision-makers, VOICES) indicated that the amount of citizen engagement in their research (or related projects/activities) had decreased.

VOICES members at follow-up reported that they “felt more a part of the larger TREC family”. Decision-makers and the research team stated that there was greater visibility and presence of VOICES members at team meetings. Numerous respondents indicated that the priority setting exercise was an important and useful strategy to engage VOICES members to provide direction for the research team. A trainee noted that even though several activities had occurred to integrate VOICES to the team, there were still opportunities for more meaningful engagement from an early stage of research training.

“From a trainee perspective, I don’t see much of VOICES outside of the major meetings. I wonder if there is an opportunity to connect specific trainees to VOICES members that are relevant to their project and have an interest in the topic? A sort of citizen mentorship program” (Trainee, follow-up).

## Discussion

Over 15-months, the TREC research team undertook activities to strengthen engagement with its citizen members. We administered one survey at baseline and one follow-up survey with the intention of capturing changes in the use and perceptions of citizen engagement in health research. Between baseline and follow-up, we found that a greater proportion of respondents reported being engaged in research, perceiving that the team had the skills to conduct citizen engaged research, and being able to identify roles for citizens in the most stages of the research process. Despite the perceived benefits of citizen engagement at each time point, which were many, challenges persisted, many specifically related to the practical methods of building citizen engagement into regular research practice.

### Benefits of engagement

Respondents were generally positive about including citizens in their research and they reported actively doing so in various capacities. At follow-up, a larger percentage of respondents reported participating in citizen engagement activities and reported positive perceptions of engagement relative to baseline. In the time between the baseline and follow-up survey, several strategies within TREC were introduced to improve engagement (including ‘patient engagement’ training and normalizing the presence of citizen partners at research meetings). Perhaps the most notable strategy that was referenced by respondents in the survey was their positive perception of the priority setting exercise that was used to engage citizens and stakeholders to identify research questions and priorities for secondary data analysis [16]. Respondents valued this activity as a way to engage citizens in the research and this could have contributed to the higher proportion of respondents indicating that a role for citizens in research was to identify research questions.

One of the most frequently cited benefits of citizen engagement was how they made the research more meaningful and relevant. Respondents commented on how engagement gave research greater authenticity or relevance to real world issues, like the perceived “validation effect” reported by Thompson et al. [19]. However, research on the impact of engagement on research quality or outcomes and evidence in this area continues to be sparse. To date, there has been little study of the extent to which engagement leads to more relevant research to end-user needs or quicker uptake of findings in practice and what engagement activities are most relevant to the uptake of research. This points to a consistent dilemma with the proliferation of citizen engaged research. Including citizens can make the work more enjoyable and meaningful and the fact that it feels good has become an increasing justification for engaging citizen partners; however, this must be considered alongside the concerns from respondents about, often unintentionally, perpetuating tokenism and overextending partners. Citizen inclusion in research must be balanced with continued education, training, and critical reflection. Even after a year of intentional integration efforts, respondents still noted that they required guidance on communication and other basic operational areas to carry out studies that meaningfully include citizens.

### Need for support

We found that respondents’ perceptions of challenges primarily related to the practical aspects of engagement and these perceptions persisted after over a year of activities. While the team training provided a preliminary overview of citizen-oriented research and the ways in which citizens can be engaged, the training did not truly address the structural challenges expressed by many of the research team members in our survey results. Engagement, as well reported [20], requires a significant investment of both researcher and citizen time. This phase of relationship building and integrating citizens in the early stages of research often occurs before a researcher has obtained grant funds [21]. Activities related to identifying citizen partners, establishing roles and expectations, and building a meaningful collaborative relationship are not considered in traditional academic merit systems (i.e., CV, tenure applications). This could make engaging in partnered research particularly challenging for earlier career researchers. Identifying and recruiting potential new partners, building meaningful and trusting relationships, and carving out time to identify common research priorities all take resources that are limited. In a systematic review of the impact of engagement on researchers, community members, and others, Brett et al. [22] found similar challenges. Researchers reported difficulty undertaking meaningful engagement due to lack of funding and time; while non-academic (citizen) partners felt a lack of preparation and training for research and some felt overburdened by the demands of research [23].

As with others [24], our findings revealed that researcher respondents required more guidance on how to manage the basic administrative aspects of citizen engagement including allocating financial resources, remuneration, and staff support. Practical guides and frameworks that fit within the university administrative context are needed to assist researchers in initiating or expanding their engagement efforts. Developing and maintaining a meaningful relationship with citizen partners requires that academic teams have resources and support to adapt to the needs of their citizen partners in their research and team activities. After 15 months of activities, research team responses about challenges included the need for training and assistance with issues of accessibility and accommodations for citizen members. Researchers must be cognizant of issues of accessibility such as meeting locations, timing, materials, language, and food. Citizen partners need to be able to fully participate and not be unnecessarily challenged by the research environment. In our case, this has meant considering physical accessibility of meeting locations, covering the costs of family/friend caregivers to accompany citizen members, adjusting meeting length, and menu considerations (e.g., avoiding food items that can be dangerous for people with swallowing challenges). Such considerations often come with additional costs and require staff support to organize and implement. Researchers also need guidance and institutional support to provide sufficient renumeration to partners that recognizes the value of their time and reimbursement for services that enable citizens to engage in research.

One of the challenges noted by researchers in both surveys was identifying citizen partners. Databases developed by organizations like the provincial SPOR Patient Engagement Platforms [8] and the Alzheimer Society of Canada [25] are important resources for researchers to identity potential citizen partners. Resources like the SPOR platform are important to assist in recruitment but challenges remain if you are trying to recruit from a marginalized or stigmatized group. In our case, we initially recruited from our existing networks and asking local organizations however we quickly realized that given the national reach of the research team we needed greater representation from regions across the country. Our attempts to have representation from multiple perspectives and regions have brought diversity of experiences however it does bring challenges with different provincial health systems, diverging goals, and logistical coordination.

Once a core group of citizens are engaged, our respondents raised concerns about over-using or over-extending our VOICES members since they are known to the team and easy to connect with. Many VOICES members engage with other research teams and advocacy organizations alongside their work with TREC meaning that the potential for over-exertion is amplified further. To address the concerns about citizen fatigue, we need to continue to develop mechanisms to identify new partners and give our citizen partners the opportunity and confidence to say “no” when needed. Although not raised directly by our respondents, over-use of citizen/patient partners relates to broader concerns about “professionalization” of partners, a situation where the more citizen partners gain exposure to research and are embedded in research teams, the less they are representative of the “average” experience [24]. A study examining researcher preparedness to conduct ethical patient oriented research considered the emergence of “professional patients” the most pressing ethical issue, explaining that this occurs when a select few patients are continuously engaged in patient/citizen projects and over time these individuals begin to more closely resemble researchers rather than the patient/citizen community [24]. Continuously using the same partners may ease the job of researchers but ultimately limits diversity of citizen experience. Ives et al. [26] echo this concern and add that the benefits of patient engagement depend on the ability of the patient to bring the “outsider” perspective rather than being immersed in the research world. The ability to recruit new members, while sustaining the relationships, and capacity that we have built with the existing team is an ongoing operational and ethical challenge.

In our research we found that the available training programs and guiding frameworks for partnered research still appear to be lacking even as the amount of engagement has increased. The need for training for both researchers and non-academic partners—with different goals for each—was raised several times, in particular the need to understand each other’s roles, agendas, managing power imbalances, and expectations for timelines and research impacts. For researchers training on communication, strategies to keep partners feeling engaged while awaiting funding and over the course of a study, and ideas on when, if and how to incorporate partners into the more technical phases of the research were needed. Even with the training programs provided by SPOR and other organizations, there continues to be a lack of clear practical information on the steps to making engagement successful [8]. The team training that we received from our SPOR support unit was led by trained external facilitators and did not meaningfully integrate the citizen members. Future iterations of the training program should consider a co-created training experience that involves citizens in the training and offers more practical strategies for teams wherever they are in their engagement process. To some extent, this is like the challenges in the field of knowledge translation (KT). McLean et al. [21] found that while KT efforts increased due to incentives from research funders, there were no standard approaches or mechanisms to evaluate or support these efforts. Citizen engagement is at risk of being one in the same. Funders express their interest and then requirement for engagement; yet, the support for the implementation and evaluation of these efforts is piecemeal at best. If we are to include citizen partners on our teams, taking their time and intellectual and emotional resources, we must do more to apply evidence based approaches and monitor these efforts so that the benefits of engagement can be realized in health research.

## Limitations

Our study focused on a single research team that has a history of engagement with non-academic stakeholders. Therefore, the team may be more open and/or prepared to engage with citizen partners than other research teams. We had a good response rate to our baseline survey, but our response rate declined for the follow-up survey. We had respondents in the baseline survey who did not report their role and we were unable to attribute their results to a specific group. Since surveys were anonymous, we could not assess non-response bias nor could we determine how many people completed both a baseline and follow-up survey. It is possible that any increase in reported engagement activity and/or perceived benefits resulted from fewer responses to the follow-up survey from team members with less interest or investment in engagement. Therefore, our results to the follow-up survey may reflect only those with a deeper interest in engagement. In that case, the reported challenges and needs for support would likely be stronger among non-respondents who require even more guidance to conduct engaged research. Finally, while our study assessed a single research team’s experiences with and perceptions of engagement, we are unable to generalize beyond this teams experience and were unable to evaluate the impact of engagement on the team’s research or its uptake. This is an area that continues to lack guidance and research.

## Conclusion

Engaging people with lived experience has come to be an expectation in health research. While engagement is generally received positively, challenges related to adequate training, funding, and other organizational supports are common and persist even as researcher and non-academic team members gain exposure to different aspects of engagement. Our survey results indicate that researchers remain unsure if and how citizens can be engaged in various research activities. This, combined with a lack of evidence on the impact of engagement on research quality and the potential outcomes, suggests that work is still needed to understand how to gain the most from citizen engaged research.

## Supplementary Information

**Additional file 1**. “Baseline survey” and “Followup survey”.

## Data Availability

The data generated during this study are available from the corresponding author on reasonable request.

## Abbreviations

TREC: Translating research in elder care
VOICES: Voices Of Individuals, family and friend Caregivers Educating uS
SPOR: Strategy for patient-oriented research
PCORI: Patient centered outcomes research institute
