# Supplementary material for: Evolving partnerships: engagement methods in an established health services research team
Source: Res Involv Engagem. 2021 Oct 9;7:71. doi: 10.1186/s40900-021-00314-w (PMC8502285; doi:10.1186/s40900-021-00314-w)
Supplement: Supplementary file 1 — Additional file 1. “Baseline survey” and “Followup survey”. [file 40900_2021_314_MOESM1_ESM.docx]

Supplementary Files

Baseline Survey (May 2018)

**What is your role with TREC?**

- Decision-Makers
- Investigators
- Staff
- Trainees
- VOICES
- Other

**I am currently involved in a research project that includes citizen partners.**

- Yes
  - If yes, please provide a short description of each project and indicate it is a TREC project
- No

**How familiar are you with the citizen engagement spectrum? [insert spectrum graphic]**

- Not at all familiar
- Slightly familiar
- Somewhat familiar
- Moderately familiar
- Extremely familiar

**Of the engagement methods described in the figure above, which ones are you using in your CURRENT research projects? (can select multiple)**

- Learn/Inform
- Consult
- Involve
- Collaborate
- Empower/Lead
- Not currently involved in research with a citizen partner, but have been previously
- I have not engaged in any citizen engagement activities

**Of the engagement methods described in the figure above, which ones are you using in your PREVIOUS research projects? (can select multiple)**

- Learn/Inform
- Consult
- Involve
- Collaborate
- Empower/Lead
- Not applicable

**The research cycle consists of 5 categories of activities. Do you see a role for citizen partners in the following phases of the research cycle? (Can select multiple)**

- Identifying the research question(s) and objective(s)
- Designing the study
- Collecting the data
- Analyzing the data
- Dissemination the findings

**How can a citizen be involved in this (selected) stage?**

**What do you see as the potential benefits of citizen engaged research? (can select more than one)**

- Satisfy requirements for funding agencies (e.g., CIHR)
- Unique perspectives
- Support for knowledge translation
- Generate new ideas
- Provide connections to other relevant groups (other citizen groups, decision-makers, front line staff)
- Access/support for data collection (e.g., connection to new sites, assist in recruiting participants)
- I do not see a benefit to partnered research
- Other (please specify)

**What do you see as the challenges of citizen engaged research? (can select more than one)**

- Time
- Cost
- Identifying citizen partners
- Loss of control
- Impact on scientific rigor
- Unclear what activities partner could be engaged in
- I do not see any barriers or challenges to partnered research
- Other (please specify)

**Would you like to conduct research with citizen partners (with TREC or elsewhere)?**

- Yes
- No
- Unsure
- Already involved in citizen engaged research

**Have you previously participated in citizen engagement training?**

- Yes
- No

**Describe/provide a brief description of the training (e.g., organization/group who facilitated, format (in-person, online))**

**What support do you need to conduct engaged research? (Can select more than one)**

- Communication with non-academic audiences (written materials)
- Communication with non-academic audiences (verbal/presentation skills)
- Meeting facilitation
- Budget supports (how to budget for citizen partners)
- Staff support
- Other

**Do you feel you have the skills to conduct citizen engaged research?**

- Yes
- No
- Unsure

**What skills do you feel you need but do not currently have?**

**The VOICES members are adequately engaged in the TREC team**

- Strongly disagree
- Disagree
- Neutral
- Agree
- Strongly agree

**How would TREC improve the engagement with VOICES members? Give specific examples**

**The decision makers are adequately engaged in the TREC team**

- Strongly disagree
- Disagree
- Neutral
- Agree
- Strongly agree

**How would TREC improve the engagement with decision maker members? Give specific examples**

Follow-up (JULY 2019)

**What is your primary role in TREC?**

- Decision-Maker
- Investigators
- Staff
- Trainee
- VOICES
- Other

**Did you attend the citizen engagement training session last year (on June 13, 2018 before the 2018 TREC AGM)?**

- Yes
- No

**Since that time, whether you attended the training session or not, would you say that the amount of citizen engagement in your research (or related projects) has...**

- Increased
- Decreased
- Stayed the same

**In the past year, have you included citizen partners in any of the following activities? (response categories: yes, no, don’t know, not applicable)**

- Research priority setting
- Grant proposal/protocol writing
- Research question development
- Study design development
- Data collection
- Data analysis
- Interpretation of results
- Knowledge translation product development
- Dissemination of research findings
- Determining future research

**I am currently involved in a research project that includes citizen partners.**

- Yes
- No

**Please provide a short description for each project and indicate if it is a TREC project.**

**Of the engagement methods described in the figure above, which ones are you using in your CURRENT research projects? (can select multiple)**

- Learn/Inform
- Consult
- Involve
- Collaborate
- Empower/Lead
- Not currently involved in research with a citizen partner, but have been previously
- I have not engaged in any citizen engagement activities

**The research cycle consists of 5 categories of activities. Do you see a role for citizen partners in the following phases of the research cycle? (can select multiple)**

- Identifying the research question(s) and objective(s)
- Designing the study
- Collecting the data
- Analyzing the data
- Disseminating the findings

**Since the citizen engagement training in June 2018, have you seen or noticed a change in the extent to which VOICES members are engaged in TREC activities?**

- Yes, an increase
- No change
- Don’t know

Additional comments on what you have seen

**VOICES members have been adequately engaged in the TREC team.**

- Strongly Agree
- Agree
- Neutral
- Disagree
- Strongly Disagree

**How would TREC improve engagement with VOICES members? Give specific examples**

**Decision-makers have been adequately engaged in the TREC team.**

- Strongly Agree
- Agree
- Neutral
- Disagree
- Strongly Disagree

**How would TREC improve engagement with decision-makers? Give specific examples**

**What do you see as the potential benefits of citizen engaged research? (can select more than one)**

- Satisfy requirements for funding agencies (e.g., CIHR)
- Unique perspectives
- Support for knowledge translation
- Generate new ideas
- Provide connections to other relevant groups (other citizen groups, decision-makers, front line staff)
- Access/support for data collection (e.g., connection to new sites, assist in recruiting participants)
- I do not see a benefit to partnered research
- Other

**Based on what you have seen through TREC's engagement over the past year, is there anything that you would now consider a benefit of citizen engagement for research that you did not previously?**

**What do you see as the challenges of citizen engaged research? (can select more than one)**

- Time
- Cost
- Identifying citizen partners
- Loss of control
- Impact on scientific rigor
- Unclear what activities partner could be engaged in
- I do not see any barriers or challenges to partnered research
- Other

**Over the last year, is there anything you would now consider a challenge to including citizens in your research?**

**What support do you need to conduct engaged research?**

- Communication with non-academic audiences (written materials)
- Communication with non-academic audiences (verbal/presentation skills)
- Meeting facilitation
- Budget supports (how to budget for citizen partners)
- Staff support
- Other

**Do you feel you have the skills to conduct citizen engaged research?**

- Yes
- No
- Unsure

Do you feel you have developed skills to conduct engaged research based on the patient engagement training (June 2018) and/or your experiences through TREC?

- Yes
- No
- Unsure

**What skills do you feel you need but do not currently have?**

**Please respond to the following statements (response categories: agree, neutral, disagree, not applicable)**

- Engaging citizens is important.
- Engaging citizens can improve the value of research.
- Engaging citizens can improve the value of the healthcare system.
- Citizens have a right to be engaged in research.
- Engaging citizens is an integral part of citizen-oriented research.
- As a researcher, citizen engagement is compatible with my program of research.
- As a researcher, I feel pressured to engage citizens in my research.
- As a researcher, engaging citizens in my research will inject bias in my research.
